# Rapid measurement of organophophorous level in serum

**DOI:** 10.4103/0019-5278.58921

**Published:** 2009-12

**Authors:** Viroj Wiwanitkit

**Affiliations:** Wiwanitkit House, Bangkhae, Bangkok Thailand - 10160

Dear Sir,

I have read the recent publication by Singh *et al*., with great interest.[1] The measurement of organophosphorous level is an important laboratory investigation in occupational medicine. Singh *et al*. reported the use of a chromatography-based technique for this purpose.[1] A good diagnostic property can be seen.[1] However, this is still a complicated technique. It needs a standard medical laboratory that poses the chromatography analyzer and requires an expert or medical technologist for performing the test. In addition, the cost of running the chromatographical analysis is high and the turnaround time starting from the specimen collection to the analysis in the laboratory with adequate facilities can be long. Rapid measurement for screening program should be less complex. Indeed, there are some alternative techniques that are more rapid for measurement. For example, Cho *et al*., reported an ELISA and ELISA dipstick technique.[2] In addition, No *et al*.,[3] also reported another new cholinesterase-based dipstick assay for the detection. These dipstick tests are more rapid that serve the analytical purpose.
